# News and Events of the Week

**Published:** 1910-11-12

**Authors:** 


### News and Events of the Week.

Mr. Abraham Hoskins, M.R.C.S., L.S.A., of The Gables, Blackroot Road, Sutton Coldfield, Warwick, surgeon, who died on October 8, aged eighty-seven, left estate valued at £47,330 gross and £45,943 net.

It is understood that the Local Government Board is preparing regulations giving power to local authorities to enforce the destruction of rats in districts suspected to have rats infected with plague, or in which their presence is reasonably suspected.

At the Royal Institute of Public Health on Monday Dr. C. Levaditi, of the Pasteur Institute, Paris, gave a lecture on "The Mechanism of Phagocytosis and the Importance of the Phagocytes in Immunity." The lecture was illustrated by cinematograph films prepared from Dr. Levaditi's preparations in collaboration with M. Comandon, of Pathé Frères. Another demonstration of these and other films, illustrating problems in bacteriology, was given at St. Thomas's Hospital on Wednesday last.

At a meeting of the General Committee of the King Edward Memorial Fund, held last Monday at the Mansion House, it was resolved that, apart from the provision of a larger memorial of his Majesty, a statue of King Edward VII. be erected in some prominent position in London, to be hereafter decided upon, and that a fund be immediately opened for the purpose. The other schemes of memorials were referred back to the executive committee. This, it was stated, would cover not only the four proposals to which the Executive Committee had already reduced the 164 hitherto considered, but such schemes as Lord Northcote's, to endow tropical research, which had been submitted too late for adequate deliberation.

Medical men will read with interest the following extract from Professor Karl Pearson's letter to the *Times* of November 8 on Heredity and Crime: "May I suggest that one of the most valuable additions that could possibly be made to the Prison Department at the present time would be the appointment of a medical man with one or two assistants, whose special occupation should be tracing the family history (chiefly from police records) and the environmental conditions in early life of convicted criminals? We should soon have sufficient material on which a definite judgment might be based as to whether crime or the tendency to law-breaking is or is not hereditary."

The vital statistics of the Victorian Year Book, just issued, show that the marriage-rate of the last five years has increased by 9 per cent. The births for the year were 31,549, and were greater than in any year since 1896. The deaths for the year (14,436) gave the rate 11.24 per 1,000—the lowest ever recorded. The infantile death-rate is steadily falling, having been 7.13 per 100 births. Of the 44,745 persons who have died in Victoria during the last three years 5,022 were aged 80 years and upwards, and nineteen of them had attained or passed the century. The average rate of natural increase per 1,000 of population for the last five years was 12.9 in Victoria and 15.5 for the whole continent, a rate higher than in Japan or any European country, except Russia, Bulgaria, and Servia.

The Coronation of his Majesty the King has been fixed to take place on Thursday, June 22, 1911, and his Majesty has appointed Tuesday, December 27 next, to be an extra Bank Holiday.

A portrait of Sir Samuel Wilks, M.D., F.R.S., President 1896-99, has been offered by the medical staff of Guy's Hospital for the acceptance of the College of Physicians. The portrait has been accepted and the thanks of the College accorded.

The Premier of Victoria (Mr. Murray) is pushing on his crusade against venereal diseases. A portion of the Melbourne Gaol not required for prison uses may possibly be set apart as a place for treatment. Dr. Konrad Hillier, of the Bacteriological Laboratory of the University, gives great help and information in this matter.

After thirty-five years' service, Mr. James Edward Barton has given up the position of medical superintendent of Brookwood Asylum, and Dr. J. A. Lowry, M.D., the first assistant medical officer, has been appointed as his successor. The vacancy caused by Dr. Lowry's promotion has been filled by Mr. P. C. Coombes, who was second assistant medical officer at Netherne Asylum, Merstham.

Graces were offered to the Senate of the University of Cambridge on Thursday last proposing that the offer from the Drapers' Company of a new physiological laboratory be gratefully accepted, and that the Vice-Chancellor, the Master of Pembroke, the Master of Christ's, Dr. Langley, Professor of Physiology, Dr. W. M. Fletcher, and Mr. K. Lucas, M.A., Trinity, be the Syndicate to discuss details with the Company.

At a meeting of the King's College Hospital Medical Society, held last week at King's College Hospital, there was an exhibition of films by Messrs. Pathé Frères showing the possibilities of the cinematograph as applied to microphotography. This was the first time these films had been shown in London. This application of cinematography appears to be valuable to medical teaching, since by its aid a preparation which by ordinary methods can be viewed only by a single student in a microscope can be displayed to a large class.

The seventh International Congress of Dermatology and Syphilography will be held at Rome from September 25 to 29, 1911, under the presidency of Professor T. de Amicis, of Naples. Dr. Gaetano Ciarrochi, Piazza Grazioli 5, Rome, and Dr. Luigi Silvestri, 13 Via della Pace, Rome, will act respectively as secretary and treasurer. The subscription has been fixed at £1. Communications must be announced to the committee before April 30, 1911. The following questions have been announced for discussion: (1) What influence have recent etiological, diagnostic, and experimental researches exerted upon the direction of the treatment of syphilis, the possibility of immunisation, and the possibility of radical or abortive treatment of the infection? (2) The results of physicotherapy in skin diseases. (3) Blastomycosis sporotrichosis and their relations with analogous processes.

The Pope last Saturday received his oculist from Dublin, who tested his Holiness's sight, and found that it had scarcely changed since the examination made in 1908.

Professor Osler will deliver a lecture on November 17, at five o'clock, in the large theatre of the Cambridge University Medical Schools, on "Medical Education in France."

The number of medical students who have entered the University of Cambridge this year is ninety-eight. Compared with the numbers that entered last year and in 1908 this shows a decrease of twelve and fifteen respectively.

The following are the arrangements for the week at the Central London Throat and Ear Hospital, Gray's Inn Road, W.C.—Lectures : Tuesday, November 8, at 3.45 p.m., "Nose," by Dr. Atkinson; Friday, November 11, 3.45 p.m., "Labyrinth," by Dr. Dan McKenzie.

Dr. William Thomas Law, M.D., F.R.C.S., of Dun-Kellin, Crabton Close Road, Boscombe, Bournemouth, one of the pioneers of the open-air treatment of phthisis and the author of several medical treatises, who died on September 6, aged sixty-five, left estate valued at £8,950 gross, and £8,536 net.

Richard Middlemore Post-Graduate Lectures, 1910.—The annual lecture under this endowment will be delivered at the Birmingham and Midland Eye Hospital by Mr. Sydney Stephenson, D.O. (Oxon.), ophthalmic surgeon to the Queen's Hospital for Children, London, etc., etc. Subject, "The Consequences of Eye-Strain," Thursday, December 1, at 4 p.m.

The Lord Mayor has become Chairman of a Mansion House Committee which has been formed to promote the raising of upwards of £30,000 for the erection of a new building for the Royal Society of Medicine. The new site is at the Cavendish Square end of Wimpole Street. Towards the money in hand £8,500 has been subscribed by members of the medical profession. The Society now has 3,200 Fellows and members and possesses a library of nearly a hundred thousand volumes. It was originally founded in 1805, under the name of "The Royal Medical and Chirurgical Society," and, as will be remembered, a new charter was granted it in 1905 under its present name.

In view of the interest excited in the question of London's ambulance service, the following remarks from Admiral W. H. Henderson in a letter to the *Times* are worth quoting. The Admiral is Chairman of the Ambulance Committee of the Metropolitan Asylums Board : "The Metropolitan Asylums Board possess a complete infectious and non-infectious service, which is being converted from horse to motor traction, with the necessary organisation for maintenance and repair. Had the Asylums Board been entrusted with it, I have no hesitation in saying a complete service would have been in full working order long before now, provided at a less cost than if undertaken by another authority having to duplicate many points of a service already in existence. An ambulance service for all purposes is a necessity which it is the duty of a public authority to provide; it cannot be done by private enterprise; and it is obvious, if economical efficiency is to be secured, only one authority should be responsible for all ambulance work."

The Hospitals Committee at the recent meeting of the Metropolitan Asylums Board reported that the metropolitan borough councils had been empowered, under an Order of the Local Government Board, to provide a supply of anti-toxic serum for the poorer inhabitants of their districts, and its recommendation that application be made to the Local Government Board for powers to enable the managers to supply the councils with anti-toxic serum was adopted.

An effort has been made recently at Elstead, near Godalming, to give practical effect to the work of the medical inspection of school children which is carried out under the supervision of the Surrey County Education Committee. After children had been examined hitherto nothing was done in many cases beyond the reporting of the fact to the parents. Mr. Curwen, the rector of Elstead, has organised a committee, which engaged the services of two doctors, who in the village hall, temporarily converted into a hospital, performed operations on about a dozen children. One-half the cost is being defrayed by public subscription, the other half by the parents.

The Emeritus Harveian Librarian (Dr. J. F. Payne) has presented to the library of the Royal College of Physicians some very rare editions from the press of the early English printer Pynson : *Galen's De Naturalibus Facultatibus*, T. Linacro interpr., 4to. (1523), dedicated to Archbishop Warham; *Galen De Pulsuum Usu*, T. Linacro interpr. (1522), dedicated to Cardinal Wolsey; and *Galen De Motu Musculorum* (1522), translated by N. Leonicenus and edited by Linacre. The first Greek edition of Plutarch's *Lives*, printed in 1517, a copy which bears the inscription "T. Linacri" in the handwriting of the Founder, has been added to the library. It came from the library of the Throckmortons at Weston, where it had been since 1684. It is a fine copy in the original binding.

